# Model-based clustering reveals vitamin D dependent multi-centrality hubs in a network of vitamin-related proteins

**DOI:** 10.1186/1752-0509-5-195

**Published:** 2011-12-02

**Authors:** Thanh-Phuong Nguyen, Marco Scotti, Melissa J Morine, Corrado Priami

**Affiliations:** 1 The Microsoft Research - University of Trento Centre for Computational and Systems Biology (COSBI), Piazza Manifattura 1, 38068 Rovereto (Trento), Italy; 2 Department of Information Engineering and Computer Science, University of Trento, Via Sommarive 14, 38123 Povo (Trento), Italy

## Abstract

**Background:**

Nutritional systems biology offers the potential for comprehensive predictions that account for all metabolic changes with the intricate biological organization and the multitudinous interactions between the cellular proteins. Protein-protein interaction (PPI) networks can be used for an integrative description of molecular processes. Although widely adopted in nutritional systems biology, these networks typically encompass a single category of functional interaction (*i.e*., metabolic, regulatory or signaling) or nutrient. Incorporating multiple nutrients and functional interaction categories under an integrated framework represents an informative approach for gaining system level insight on nutrient metabolism.

**Results:**

We constructed a multi-level PPI network starting from the interactions of 200 vitamin-related proteins. Its final size was 1,657 proteins, with 2,700 interactions. To characterize the role of the proteins we computed 6 centrality indices and applied model-based clustering. We detected a subgroup of 22 proteins that were highly central and significantly related to vitamin D. Immune system and cancer-related processes were strongly represented among these proteins. Clustering of the centralities revealed a degree of redundancy among the indices; a repeated analysis using subsets of the centralities performed well in identifying the original set of 22 most central proteins.

**Conclusions:**

Hierarchical and model-based clustering revealed multi-centrality hubs in a vitamin PPI network and redundancies among the centrality indices. Vitamin D-related proteins were strongly represented among network hubs, highlighting the pervasive effects of this nutrient. Our integrated approach to network construction identified promiscuous transcription factors, cytokines and enzymes - primarily related to immune system and cancer processes - representing potential gatekeepers linking vitamin intake to disease.

## Background

Nutritional systems biology is an emerging field that aims to characterize the molecular link between diet and health in an integrated fashion [1]. Interactome models, in particular protein-protein interaction (PPI) networks, are fundamental to nutritional systems biology in providing an abstraction of the complex relationships between molecular components - ranging from nutrients and their derivatives to diet-sensitive transcription factors. To date, the majority of network-based studies in nutritional systems biology have focused on a single interaction paradigm - *i.e*., metabolic, signaling or regulatory. However a systems biology-oriented approach should incorporate multiple parallel cellular processes [2]. In the case of nutritional systems biology, this approach entails integrated analysis of nutrient metabolism along with nutrient-mediated activation of gene expression and signaling cascades.

Vitamins are an appealing dietary component to be studied under such an integrated framework, as they comprise a heterogeneous group of organic compounds that affect a wide range of metabolic, signaling and regulatory processes. For example, vitamin B12 acts as a cofactor for a number of isomerases and methyltransferases [3], whereas vitamin C and E have well-studied antioxidant function [4]. Vitamin D serves a hormone-like function, affecting gene transcription through activation of the vitamin D receptor [5]. The degree to which the molecular effects of diverse vitamins overlap and intersect has been assessed in a reductionist way in several studies on vitamin synergy [6-8] but has yet to be assessed in a holistic, inclusive fashion.

An intriguing question in the analysis of biological networks is whether topological prominence of a protein implies biological importance. Some studies have emphasized how well-connected hubs seem to be of high functional importance [9-11]. Zotenko *et. al*. found that essentiality is due to the involvement of hubs in essential complex biological modules, groups of densely connected proteins with shared biological function that are enriched in essential proteins [12]. This connection between centrality and functional importance is complicated by the multitudinous approaches for measuring these indices [13,14]. del Rio *et al*. argued that the combination of at least two centrality measures allows to predict essential genes from molecular networks [15]. This perspective poses serious concerns on the minimum and optimal set of centralities that are needed to characterize functional properties of the network nodes (*e.g*., proteins, genes). Although redundancy among centralities has been investigated in social networks [16], food webs [17] and landscape networks [18], there is a lack of insight about their correlations in biological networks.

In this work, we obtained 200 proteins linked to vitamins (vitamin proteins, in short) by mining all human protein data published in the Universal Protein Resource (UniProt) database [19]. These proteins span a range of biological functions, including metabolic enzymes, signaling proteins, nuclear receptors and transcription factors. Based on the initial list of vitamin proteins, we mined all first degree neighbors of the vitamin proteins from the Interologous Interaction Database (i2d) [20], resulting in an integrated network of metabolic, signaling and regulatory proteins and their immediate interactions. We then estimated 6 centralities, characterizing each protein at the local, intermediate and global scale, and applied model-based clustering to identify the high-centrality network hubs. Furthermore, we assessed the centrality indices to determine the degree of unique information provided by each index.

## Methods

### Network construction

We considered two databases in constructing the network: the Universal Protein Resource and the Interologous Interaction Database. The UniProt database is the most comprehensive, high-quality and freely accessible resource of protein sequence and functional information. It is composed of 525,997 entries - version March 2011. The i2d is an online database of known and predicted mammalian and eukaryotic protein-protein interactions. It includes 482,388 relationships (111,229 human interactions) - version 1.8. The data under investigation are human-specific.

We extracted all human proteins that are related to vitamins; all published information was manually checked to identify its relatedness to vitamins. In the UniProt database, vitamin-associated information is found in different types of data such as biological function, processes, reference databases and keywords. For example, protein Q13111 (chromatin assembly factor 1 subunit A) is described as functionally related to vitamin D. It is involved in vitamin D-coupled transcription regulation via its association with the vitamin D receptor (VDR). Certain specific keywords in the UniProt database consist of vitamin-related information, but they are not presented explicitly. Protein Q13085 (AcetylCoA carboxylase 1) is classified with the functional keyword "Biotin", a member of the B complex vitamins essential for fatty acid biosynthesis and catabolism. It also acts as a growth factor for many cells and its synonyms are vitamin B7, vitamin B8, vitamin H, Coenzyme R, Biopeiderm (see more at http://www.uniprot.org/keywords/KW-0092). Note that we excluded all proteins that are not yet reviewed by UniProt curators. With this approach, we obtained a set of 200 vitamin-associated proteins (Additional file 1). Direct interactions involving the 200 proteins were extracted from the i2d. This search retrieves 6,361 protein-protein interactions. Some of these interactions are redundant as they are obtained from different datasets, or predicted by homologous methods. To increase the confidence in the interaction dataset, we excluded all the interactions inferred through homology. The resulting vitamin-related PPI network is composed of 1,705 proteins and 2,700 interactions. The network is binary (all interactions are unweighted) and undirected. We performed our analyses on the giant component (the connected sub-network that includes the majority of the entire network proteins), which contained 1,657 proteins and 2,672 interactions (Additional file 2).

### Network analysis

To describe the global properties of the network we measured density (the ratio between the number of interactions and the number of possible interactions), clustering coefficient (the probability that the adjacent proteins of a protein are connected), diameter (the length of the longest shortest path between two proteins) and average path length (the average number of steps separating all possible pairs of proteins via shortest paths).

We characterized the biological importance of proteins using indices of topological centrality. Many studies demonstrate the presence of strong correlations between the PPI network structure and the functional role of its protein constituents [9,13,21]. Since each centrality describes a unique structural feature, reliable predictions of the biological properties can be achieved by combinations of these measures, rather than relying on a single index [15]. In this study we analyzed centralities related to local (degree and eigenvector scores), intermediate (topological importance up to 1 and 4 steps) and global (betweenness and closeness) scale.

Degree (*D*) quantifies the local topology of each protein, by summing up the number of its adjacent proteins [22]. An alternative measure of local importance is represented by eigenvector scores of network positions (*EC*) [23]. These scores depend on a reciprocal process in which the value measured for a protein is proportional to the sum of the scores of its neighbors. While degree centrality gives a simple count of the number of interactions of a given node, eigenvector centrality is based on how influential are the neighbors, weighting their interactions. In general, highest scores are computed for proteins that are connected to many other proteins within large cliques or high density clusters.

The topological importance (*TI*) considers the spread of indirect effects at a meso-scale level [24]. It is based on the relative number of interactions linking a target protein to surrounding proteins, in comparison to the complete arrangement of interactions (direct or indirect) among the surrounding proteins. This index is derived from the analysis of two-step long, horizontal, and apparent competition interactions in host-parasitoid networks [25]. When the vertex *i *can be reached from *j *in *m *steps, the effect is defined as *r_m,ij_*. In presence of unweighted networks, the simplest case is with a one-step effect of *j *on *i *(*m *= 1), when the *r_m,ij _*effect equals the reciprocal of degree centrality (*r*_1*,ij *_= 1/*D_i_*, with *D_i _*= degree of node *i*). Indirect effects are multiplicative and additive. Consider, as an example, the case of a vertex *j *connected to *i *with a couple of pathways passing through *k *and *h*. The effect of *j *on *i *through *k *is defined as the product of direct effects: *r*_1_*,_kj_*·*r*_1*,ik*_. Similarly, the effect of *j *on *i *through *h *is estimated as *r*_1,*hj*_·*r*_1,*ih*_. To determine the total effect of *j *on *i*, via the two-step pathways, the additive principle is adopted: *r*_2*,ij *_= *r*_1*,kj*_·*r*_1,*ik *_+ *r*_1*,hj*_·*r*_1*,ih*_. The effect generated by *i *over *m*-steps is summarized by *φ_m,i_*.

(1) $$\varphi_{m,i}=\underset{i\neq j}{\sum }r_{m,ji}$$

$TI_{i}^{m}$ quantifies cumulated effects of a single vertex *i *on all the others in the network, up to a maximum pathway length of *m *steps. The sum of effects is normalized by the maximum number of steps considered.

(2) $$TI_{i}^{m}=\underset{q\in m}{\sum }\frac{\varphi_{q,i}}{m}=\underset{q\in m}{\sum }\underset{i\neq j}{\sum }\frac{r_{m,ji}}{m}$$

In this study we measured topological importance for direct interactions (*TI*^1^) and for proteins that lie 4-steps away from the target (*TI*^4^). We computed the topological importance up to distances of 1 and 4 steps for filling the gap between local and global centralities. *TI*^1 ^is a short range extension of the degree, while *TI*^4 ^provides a measure of the meso-scale effects at a barycentric level (consider that the diameter of the vitamin PPI network - *i.e*., the longest distance separating two proteins via shortest paths - is 11)

Betweenness (*B*) and closeness (*C*) are classical indices borrowed from social network analysis. They define the role of proteins as emerging from the relative position at the whole network level and are based on the concept of network paths. Betweenness measures how frequently the shortest path connecting every pair of proteins is going through a given protein [26]. Closeness of a protein is defined by the inverse of the average length of the shortest paths to access all other proteins in the network [22]. The larger the value, the more central is the protein.

**Figure 1:**
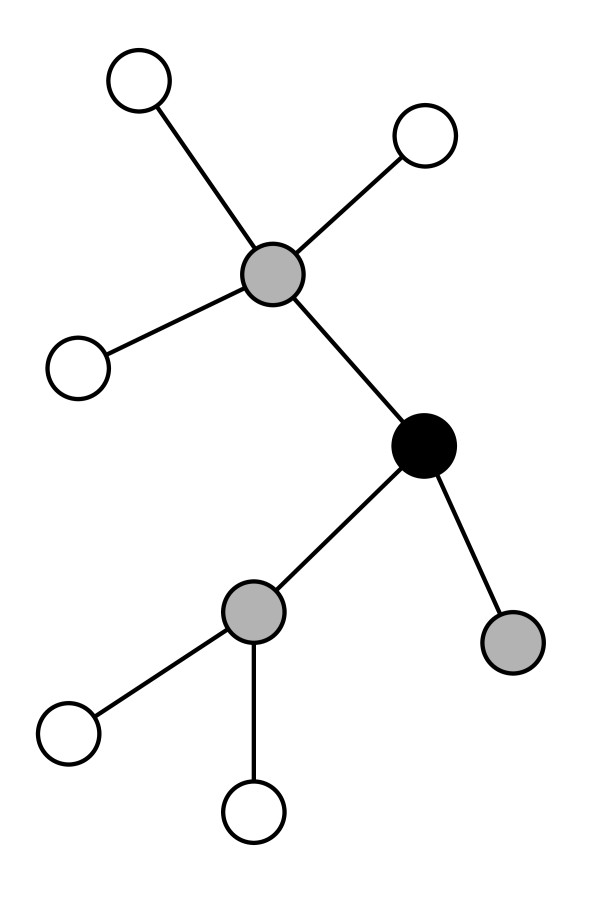
**Centralities of a target protein in a toy model**. Illustration of degree (*D*), eigenvector score (*EC*), topological importance up to one step (*TI*^1^), betweenness (*B*) and closeness (*C*). The black protein has three gray neighbors: *D *= 1+1+1 = 3. When the importance of direct connections is weighted, the black node is ranked 2nd (*EC *= 0.925) since two gray nodes out of three are highly connected (*EC *= 1.000 and *EC *= 0.676). We also know the white neighbors of its gray neighbors: *TI*^1 ^= 1 + 0.25 + 0.33 = 1.58. This latter index defines the relative importance of the target protein (in black), in comparison to the "clouds" of (white) proteins connected to its direct (gray) neighbors (since it is computed up to 1 step, *TI*^1^). The relative importance of the black protein at the whole network level depends on its mediator-role in connecting every other pair of proteins (*B *= 19), or is measured in terms of average proximity to the others (*C *= 0.615). Because of its barycentric position, the black node ranks 1st both in terms of betweenness and closeness.

We computed network centralities using Graph (Graph, COSBI, The Microsoft Research - University of Trento Centre for Computational and Systems Biology, http://www.cosbi.eu/index.php/solutions/cosbi-lab/solutions-graph) [27] and the igraph package [28]. Network visualization was realized using the software Cytoscape [29]. In Figure 1 we depicted a hypothetical network to illustrate the definition of centrality indices.

### Statistical analysis

Since centralities showed different ranges of variation (*e.g*., the maximum hypothetical degree of a target protein corresponds to the total number of the other proteins, while eigenvector scores are automatically scaled to have a maximum value of one), we made them comparable by setting the upper limit of each index to one. For identifying the most central proteins we grouped the nodes through cluster analysis; the composition of clusters is based on the centrality scores of each protein. Proteins are characterized through 6 indices of centrality which portray topological properties from the local level to the global scale. We adopted a model-based clustering (MBC) procedure using the R package mclust [30-32]. The optimal model and number of clusters were inferred according to the Bayesian Information Criterion (BIC) [33,34].

We applied the Kolmogorov-Smirnov test to measure the independence of the network structure from the current knowledge on vitamins (*i.e*., by comparing the number of manuscripts published on vitamin proteins to their degree distribution), and determining whether the most central proteins (extracted through cluster analysis) significantly deviate from the initial list of 200 (used as a reference for constructing the PPI network). With the chi-squared test we investigated differences related to (fat vs. water) solubility and involvement into the regulation of transcription. Vitamin associations of proteins in different organisms (*Homo sapiens*, *Mus musculus*, *Saccharomyces cerevisiae *and *Escherichia coli*) were compared with the Kolmogorov-Smirnov test (alternative hypothesis: two-sided). Individual vitamin associations and number of published manuscripts were extracted from the UniProt database.

To identify which centralities provide redundant information we compared protein rank orders, based on each centrality index, by adopting the Goodman-Kruskal's lambda [35]. Correlation coefficients were used to construct a dendrogram of similarities between the different indices. We repeated the same analysis to investigate redundant centralities in the case of null models that were assembled using the vitamin PPI network as a reference. Finally, we tested the performance of smaller subsets of centralities (composed of 4 indices) to determine whether the same proteins could be identified as with the full set of centralities.

All statistical analyses were performed with R [36].

## Results

The giant component of the vitamin PPI network is composed of 1,657 proteins and 2,672 interactions. This network is sparse (density = 0.002), with a number of interactions that is very far from the maximal that could be attained (clustering coefficient = 0.023). The majority of the proteins tend to be isolated in many short branches with many weakly interacting components. Despite this highly fragmented structure, average path length (4.182) and diameter (11) are surprisingly short, indicating that the spread of any information would quickly reach all of the system proteins.

We classified the proteins on the basis of 6 normalized values of centrality. To this end we applied model-based clustering and, according to BIC, selected the optimal clustering configuration (*i.e*., type of Gaussian model and number of clusters). From this, we extracted a small set of 22 most central proteins and observed a distribution of vitamin association that substantially deviated from that exhibited by the initial list of 200 proteins. We then measured Goodman-Kruskal's lambda to compare protein rank orders obtained with different centralities. This analysis highlighted how some centrality indices provide redundant information (*e.g*., the ranking estimated with degree and betweenness was overlapping for more than 92% of the proteins; see the row-*B*, column-*D *in Table 1). Finally, we applied our findings describing the redundancy between certain indices to investigate whether the group of 22 most central proteins could be identified with a smaller subset of centralities. We observed that different combinations of 4 centralities were sufficient to detect the 22 most central proteins, although the results did not correspond exactly with the outcomes of the original method (*i.e*., the 22 proteins were classified in two clusters of larger size).

**Table 1:** Matrix of Goodman-Kruskal's lambda values

	*D*	*EC*	*TI*^1^	*TI*^4^	*B*	*C*
*D*	1.000	-	-	-	-	-
*EC*	0.498	1.000	-	-	-	-
*TI*^1^	0.716	-0.103	1.000	-	-	-
*TI*^4^	0.954	0.108	0.350	1.000	-	-
*B*	0.928	0.412	0.671	0.905	1.000	-
*C*	0.582	0.756	-0.138	0.242	0.545	1.000

Correlation between protein rankings that are obtained with different centralities. Topological importance up to 1 step clearly deviates from all the other indices, and thus is clustered in a separate group in the dendrogram of Figure 6. This matrix helps to identify the centrality indices that are responsible for supplying similar protein rankings (*e.g*., *D *and *B*).

### Model-based clustering and protein ranking

**Figure 2:**
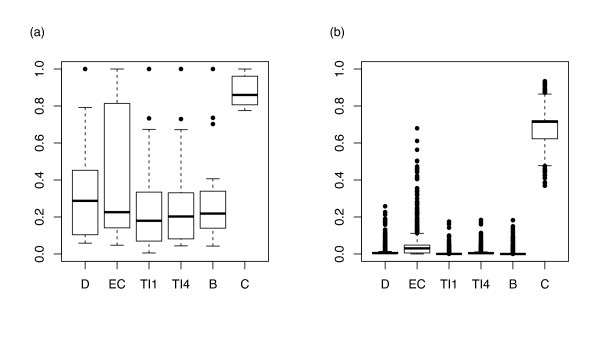
**Centrality signature of the 22 highly central proteins**. (a) Central proteins display highest average values (*μ*) for all the 6 normalized centralities (*σ *represents standard deviation): D - *μ *= 0.333, *σ *= 0.256; EC - *μ *= 0.418, *σ *= 0.347; TI1 - *μ *= 0.256, *σ *= 0.255; TI4 - *μ *= 0.271, *σ *= 0.247; B - *μ *= 0.286, *σ *= 0.243; C - *μ *= 0.881, *σ *= 0.074. (b) The remaining 1,635 proteins have average (normalized) centralities well below 0.1, except for closeness: D - *μ *= 0.010, *σ *= 0.019; EC - *μ *= 0.043, *σ *= 0.066; TI1 - *μ *= 0.002, *σ *= 0.011; TI4 - *μ *= 0.008, *σ *= 0.012; B - *μ *= 0.004, *σ *= 0.013; C - *μ *= 0.677, *σ *= 0.084. Highest scoring estimated for closeness can be explained by the short average distance between pairs of proteins in the complete network (4.182 steps).

**Figure 3:**
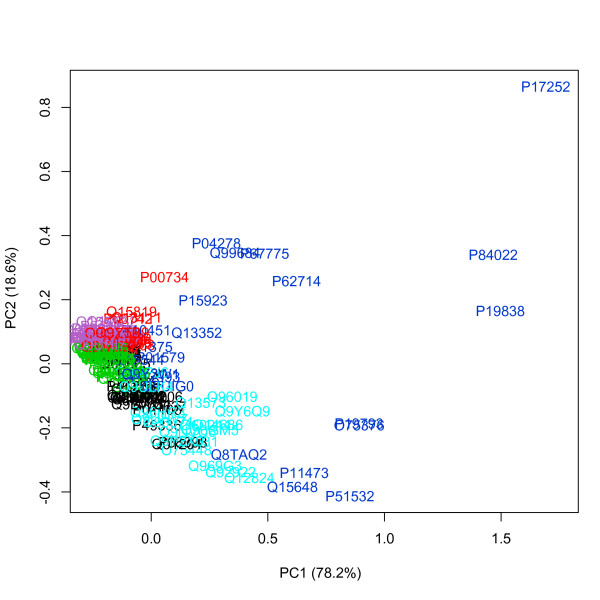
**PCA on 140 vitamin proteins (2nd round of MBC)**. Protein codes are colored based on cluster assignment from MBC, and the top 22 proteins are shown in dark blue. The two illustrated dimensions account for 96.8% of data variance, but are not associated with a single centrality. This result strengthens the utility of a multi-centrality perspective to describe the network topological properties of the proteins.

For each protein we computed 6 centralities (see Additional file 3). By assessing centrality at local (*D*, *EC*), meso-scale (*TI*^1^, *TI*^4^) and global (*B*, *C*) level we compiled a comprehensive picture of protein importance. We carried out model-based clustering for the complete set of 1,657 proteins and extracted 7 clusters, using the ellipsoidal, equal shape model (BIC = 115,732). We repeated the same analysis for further characterizing the cluster which comprised the most central proteins. The best model for its clustering was still based on the ellipsoidal, equal shape algorithm (BIC = 3,806.519), with 6 clusters. Results showed that the vitamin PPI network was centralized around a small group of 22 multi-centrality hubs. All of the most central proteins except P62993 (GRB2 - Growth factor receptor-bound protein 2) were into the initial list of 200 vitamin proteins that we used for assembling the network. The 22 proteins displayed highest average values for all the 6 normalized centralities (Figure 2); principal component analysis indicated a clear deviation from the other 118 proteins extracted after the first step of model-based clustering (Figure 3).

**Figure 4:**
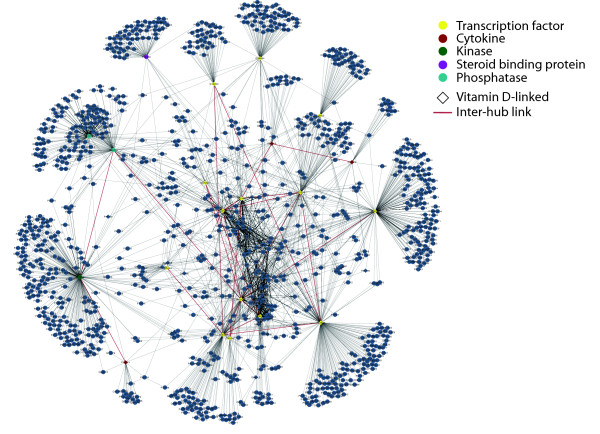
**Sub-network of the 21 highly central (vitamin-associated) proteins**. Blue neighbors are connected to central proteins via black interactions, while the connections involving two hubs are highlighted in red. Different colors refer to the specific biological roles, with vitamin D-associated proteins represented by diamond-shaped nodes. The majority of these central proteins (*n *= 17) are transcription factors, and all of them are related to fat-soluble vitamins.

All of the 21 high-centrality proteins belonging to the initial set of 200 vitamin-related proteins were linked to fat-soluble vitamins (*i.e*., vitamin D, E or K). The majority of these high-centrality proteins (17 out of 21) is involved into the regulation of transcription. Transcription-related proteins are mainly associated to vitamin D (16 out of 17), while the remaining nodes are more evenly distributed between vitamin K (P04278, SHBG - Sex hormone-binding globulin), D (P10451, SPP1 - Osteopontin) and E (P17252, PRKCA - Protein kinase C alpha type; P62714, PPP2CB - Serine/threonine-protein phosphatase 2A catalytic subunit beta isoform). These non-transcription factor nodes are classified as steroid binding protein (P04278), cytokine (P10451), kinase (P17252) and phosphatase (P62714). The structure of the sub-network involving multi-centrality proteins and their neighbors is depicted in Figure 4.

**Figure 5:**
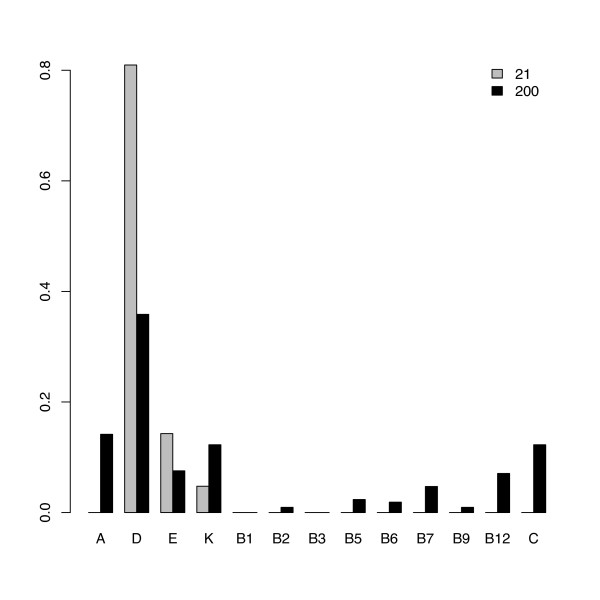
**Vitamin associations of the 21 highly central proteins**. Bar-plots describing the relative distribution of vitamin associations in the 21 most central nodes (gray) and complete list of 200 initial proteins (black). Highly central proteins are enriched with respect to vitamin D association.

We used chi-squared and Kolmogorov-Smirnov tests to compare the biological properties of multi-centrality proteins and the complete list of 200. The 21 most central vitamin proteins were significantly different from the initial 200 in terms of the proportion of proteins associated with fat-soluble vs. water-soluble vitamins (*χ*^2 ^= 65.407, *df *= 1, *p *≪ 0.001) and proportion of transcription factor proteins (*χ*^2 ^= 123.655, *df *= 1, *p *≪ 0.001). Moreover, the distribution of individual vitamin associations of the 21 key proteins deviates from the distribution observed with the full set of 200 proteins. This is mainly due to an enrichment in vitamin D-associated proteins among the central proteins, while other vitamin proteins are under-represented (see Figure 5). A significant difference is observed when considering 13 vitamins (*i.e*., keeping all the B vitamins in different classes; alternative hypothesis: one-sided - *D *= 0.615, *p *= 0.007), while it vanishes in the case of six main classes (*i.e*., by grouping all of the B vitamins; one-sided - *D *= 0.667, *p *= 0.070).

The degree distribution of vitamin proteins is significantly different from the one of manuscripts related to them (alternative hypothesis: two-sided - *D *= 0.505, *p *≪ 0.001). In case of six classes of vitamins, vitamin associations in human deviate from other organisms (mouse: one-sided - *D *= 1.000, *p *= 0.003; yeast: one-sided - *D *= 1.000, *p *= 0.003; *E. coli*: one-sided - *D *= 0.833, *p *= 0.016). Thus, our findings are not biased by the literature and are human-specific. More details are illustrated in Additional file 4. Vitamin associations of proteins for mouse, yeast and *E. coli *are summarized in Additional files 5, 6, 7.

### Goodman-Kruskal's lambda and redundant centralities

Protein rank orders based on the 6 centrality indices were compared using the Goodman-Kruskal's lambda. Each non-zero entry in Table 1 quantifies the correlations between row- and column-centralities in the vitamin PPI network.

**Figure 6:**
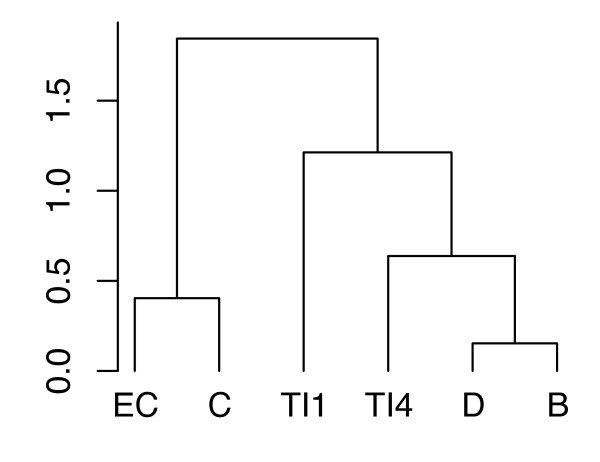
**Dendrogram of similarities between centralities in the vitamin PPI network**. Some centralities provided similar rankings, indicating a degree of redundancy of these indices. Degree and betweenness measure local and global centrality of proteins, respectively, however they provide similar protein orderings (see the Table 1; row-*B*, column-*D *= 0.928). Moreover, their patterns of correlation with the other centralities tend to overlap. Topological importance up to one step is characterized by peculiar features and is closer to *TI*^4 ^than to other indices. Closeness and eigenvector scores are grouped together.

We investigated the whole set of correlations to define which centrality indices provide redundant information. Values summarized in the correlation matrix (Table 1) were used to construct a dendrogram (Figure 6). Indices are grouped together when they provide similar protein rankings and in case of analogous relationships with the other centralities. The results showed that a similar description could be inferred by four centralities only. Indeed, we found four main groups: (a) closeness and eigenvector scores; (b) topological importance up to 1 step; (c) topological importance up to 4 steps; (d) degree and betweenness.

We carried out the the same analysis for null models that were constructed adopting the vitamin PPI network as a reference (see Additional file 8). Except for the case of rewired networks (*i.e*., the ones obtained preserving the degree distribution of the PPI network and rearranging the interactions between proteins), dendrogram structure extracted by empirical data does not match with null models.

Groupings illustrated in Figure 6 refer to the rankings measured by the 6 centralities, for the whole network. We tested whether subsets composed of 4 centralities were efficient in identifying the 22 multi-centrality hubs. As for the case with 6 centralities, we applied model-based clustering in a two-step procedure. After the first step, carried out for the whole set of 1,657 network nodes, we identified an initial group of more central proteins. A second model-based clustering was performed to further characterize this sample. Although we estimated the combinations of non-redundant centralities on the basis of the complete protein rankings, these indices were still efficient in defining the group of 22 proteins (Table 2). With two clusters we predicted the 22 most central proteins and more than the 60% of a single cluster was always composed of multi-centrality proteins.

**Table 2:** Performance of centrality subsets

	MBC_*s*1_	size_*s*1_	MBC_*s*2_	1 cluster	2 clusters
*D*-*EC*-*TI*^1^-*TI*^4^	VEV (19)	102	VVV (3)	11 (12)	19 (52)
*TI*^1^-*TI*^4^-*B*-*C*	VEV (16)	132	VVV (4)	14 (17)	22 (55)
*EC*-*TI*^1^-*TI*^4^-*B*	VEV (13)	182	VVV (4)	14 (19)	22 (68)
*D*-*TI*^1^-*TI*^4^-*C*	VEV (10)	169	VVV (4)	16 (25)	22 (63)

6 centralities	VEV (7)	140	VEV (6)	22	

Efficiency of different subsets of 4 centralities in identifying the group of 22 most central proteins extracted with the complete set of 6 indices. Topological importance up to 1 and 4 steps were always included, together with two other centralities listed in each row (*e.g*., subset of the first row = *D*, *EC*, *TI*^1^, *TI*^4^). MBC describes the optimal model obtained by clustering the proteins in the first (s1) and second (s2) step (number of clusters between parentheses). In the second column we summarized the size of the first protein cluster extracted. The number of the original 22 central proteins identified with one or two clusters are listed in the last columns (size of clusters between parentheses). VEV indicates ellipsoidal, equal shape model; VVV stands for ellipsoidal, varying volume, shape, and orientation model. The last row summarizes results obtained with the whole set of 6 centralities.

## Discussion

### Transcription factors - signaling proteins - metabolic enzymes

In the system-level view of vitamin metabolism, hubs are of central functional importance as they affect a wide range of molecular processes. The multi-level network used in this study revealed central proteins comprising multiple functional categories including transcription factors, signaling proteins, enzymes and cytokines. There were 17 transcription factors among the 21 high centrality proteins (Figure 4). Transcription factors typically induce expression of - and thus interact with - many genes, highlighting the extensive involvement of vitamins in regulation of gene expression. Similarly, protein kinases and phosphatases are enzymes that interact with diverse proteins through addition or removal of phosphate groups, resulting in alteration of target metabolic and signal transduction pathway activity. With our multi-centrality approach, the identified network hubs exhibit high centrality at the local, intermediate and global level, thus moving beyond a simple degree-based definition of protein centrality.

Since the main focus was on the importance of vitamin proteins, we constructed the PPI network including only their direct neighbors. In contrast, a network obtained with neighbors of neighbors would be less vitamin-specific and applying model-based clustering on its centrality scores would deviate from the target (*i.e*., characterizing the multi-functional backbone formed by vitamin-proteins). We adopted a pseudo ego-network perspective that well fits with the study of centralities, while networks composed by larger portions of i2d would require a more systemic view (*i.e*., the comparison between structural clusters - based on network topology - and biological clusters - that depend on the specific function of proteins; *e.g*., the proteins involved into the folate biosynthesis should be grouped together).

### Redundancy of centrality indices

Hubs in biological networks can be revealed by a mixture of topological and functional properties [14,15]. To identify most central proteins we measured local (*D*, *EC*), meso-scale (*TI*^1^, *TI*^4^) and global (*B*, *C*) indices. As previous studies demonstrated the presence of correlation between certain centrality measures [16-18], we assessed the redundancy of these 6 indices. A subset composed of 4 centralities was efficient in predicting the whole protein ranking, however there was not a perfect match with the 22 multi-centrality proteins (Table 2). This is likely due to the fact that lambda values were estimated using the whole protein rankings and not starting from the restricted group of the most important.

The dendrogram of Figure 6 illustrates how centrality indices can be clustered into 4 distinct groups. This clear partitioning may be explained by the low density of the vitamin PPI network (*i.e*., the ratio of the number of interactions and the number of possible interactions; [16]). Similar patterns are displayed by null models except for the case of small-world networks (see Additional file 8). This is because small-world networks were generated by preserving the interactions of the vitamin PPI network, but using 668 nodes (*i.e*., their density was higher than the one of vitamin PPI network and other null models). Correlations between the indices highlighted certain structural properties of the network. Degree and betweenness were classified together since the vitamin PPI network is highly assortative (*i.e*., the majority of the high-degree proteins are linked to each other; see Figure 4). This feature contrasts with previous studies, where PPI networks were characterized by high levels of disassortativity (*i.e*., hubs participated in dozens of interactions but were seldom linked by direct interactions [37,38]). We argue that this unusual backbone of highly connected hubs may relate to the way we constructed the network, in focusing only on the set of vitamin proteins and their first degree neighbors.

### High centrality of vitamin D-related proteins

Previous work has demonstrated a link between protein centrality and functional importance [39]. Cluster analysis of the vitamin proteins based on 6 centrality indices revealed 21 distinctly central nodes among the original 200 vitamin proteins. Interestingly, 17 of these hubs were linked to vitamin D, suggesting that this nutrient has pervasive effects on molecular function. Vitamin D is not considered a pure vitamin as it can be obtained both from diet and by UVB-stimulated conversion of 7-dehydrocholesterol in the skin [40]. In humans, however, sun exposure is often insufficient to meet nutritional requirements, and consequently vitamin D deficiency is considered to be an epidemic nutritional problem [41]. The vitamin D receptor (VDR) is highly specific to the vitamin D ligand, and is expressed in nearly all human cells and tissues [40]. VDR is among the most central nodes in the vitamin PPI, and also displays the property of assortativity through a large number of connections to other multi-centrality hubs in the network. This would be expected to multiply the influence of this protein on activity in the network. Accordingly, vitamin D deficiency and/or impairment of the vitamin D receptor is linked to abnormalities in bone development, hair growth, cell cycle, immune system function, glucose homeostasis and cardiovascular health [40].

In addition to pervasive involvement in molecular processes, vitamin D is proposed to have ancient origins, with vitamin D usage and VDR being conserved across diverse species of plants and animals. A common explanation for this relates to the central function of vitamin D in calcium homeostasis, an essential function in species ranging from phytoplankton to higher mammals [42]. The strong conservation of vitamin D usage and VDR may also explain the centrality of vitamin D-related proteins, as highly central proteins show a tendency for stronger evolutionary conservation than peripheral proteins [43,44].

### Functional roles of central proteins

Taken together, 17 of the 21 central proteins in the vitamin PPI formed a connected module of interactors, suggesting partially overlapping functional roles of these proteins. A number of key immune system regulators were present in this module, including the cytokines TNF*α *and IFN*γ*, the kinase KPCA and the transcription factors SMAD3, MED1, TFE2, NFKB1, RXR and VDR. The majority of these proteins are linked to vitamin D, reflecting the demonstrated molecular evidence of vitamin D intake on immune system function and widespread link between vitamin D deficiency and immune disorders [45]. The active form of vitamin D - 1,25-(OH)_2_D - stimulates production of TNF*α *in bone marrow cells through binding of a VDR-RXR complex to a response element in the TNF*α *promoter region [46]. This same complex inhibits IFN*γ *production through binding to a negative response element and interaction with an upstream enhancer element [47]. In addition to the VDR-RXR complex, uncomplexed VDR interferes with immune system regulators including NFKB1, NFAT and AP1 [48-51]. Vitamin D regulation of these critical immune system factors is expected to have widespread downstream consequences given the high centrality of these proteins in the vitamin PPI network.

In addition to immune system function, a number of the vitamin PPI network hubs play a role in cell cycle control and cancer progression (NFKB1, KPCA, SMAD3, RXR, VDR, SMCA4, TNF*α*, TFE2), reflecting previous findings that cancer-related proteins are more highly connected than non-cancer-related proteins [52]. Epidemiological studies have reported an inverse correlation between serum 25(OH)D (the 1,25-(OH)_2_D precursor metabolite) and colon, breast and ovarian cancer [53]. On a molecular level, vitamin D plays a role in cancer progression through inhibition of cell proliferation, angiogenesis and metastasis [54-56]. Among the vitamin PPI network hubs, the RXR transcription factor controls cell proliferation through dimerization with VDR and subsequent transcriptional regulation of cell-cycle related genes such as c-myc, c-fos, p21, p27 and hoxa10 [57]. Coordinated activity of these proteins is therefore critical in prevention of cancer onset and progression. Accordingly, a number of studies have demonstrated links between VDR polymorphisms and risk of a variety of cancers including skin, breast, colorectal and prostate cancer [58].

## Conclusion

The 22 proteins are multi-centrality hubs that lie in more densely connected parts of the network (*e.g*., they are characterized by highest closeness, a measure which quantifies the propensity to transmit information through direct or short paths). Moreover, they tend to interact with each other (*i.e*., high overlap between degree and betweenness; see Table 1 and Figure 6), showing many analogies with the essential proteins described by Zotenko *et al*. [12]. By using a multi-centrality approach to identifying network hubs, we have detected vitamin-related proteins that are strongly embedded in the vitamin PPI network. Given the demonstrated link between network centrality and functional importance, these proteins are expected to have pervasive effects on a range of downstream molecular processes, and thus represent potential gatekeepers in the link between vitamin intake and disease.

## Authors' contributions

TPN, MS, MM and CP conceived and designed the research. TPN, MS and MM collected and processed the data. TPN, MS, MM and CP wrote the paper. All authors read and approved the final manuscript.

## Supplementary Material

Additional file 1 **This table includes details about the initial list of 200 vitamin-related proteins**. These proteins were used as a reference for constructing the vitamin PPI network and are listed in alphabetical order. The first two columns indicate UniProtKB accession numbers and UniProtKB/Swiss-Prot entry names, while the third column describes vitamin associations, as extracted from UniProtKB. Number of publications related to each protein are shown in the last column (*source*: UniProt). The 21 most central proteins that pertain to this list are highlighted in yellow. Click here for file

Additional file 2 **Edgelist summarizing the 2,672 undirected interactions between the 1,657 proteins of the giant component**. Each line of the edgelist describes an interaction between the proteins identified by the column labels protein1 and protein2. Click here for file

Additional file 3 **This table provides the centrality values computed for the 1,657 proteins of the giant component**. Proteins are in alphabetical order and the 22 most central proteins are highlighted in yellow. Centrality indices included are: *D *= degree; *EC *= eigenvector score; *TI*1 = topological importance up to 1 step; *TI*4 = topological importance up to 4 steps; *B *= betweenness; *C *= closeness. Click here for file

Additional file 4 **For the 200 vitamin-related proteins we tested whether the number of interactions is determined by the number of publications associated with a given vitamin**. We found that the structure of the PPI network is independent from the literature. We also analyzed patterns of vitamin associations in four organisms, observing how human differs from mouse, yeast and *E. coli*. Click here for file

Additional file 5 **Vitamin associations of proteins in mouse (*Mus musculus*)**. Click here for file

Additional file 6 **Vitamin associations of proteins in yeast (*Saccharomyces cerevisiae*)**. Click here for file

Additional file 7 **Vitamin associations of proteins in *Escherichia coli***. Click here for file

Additional file 8 **Correlation matrices describing the Goodman-Kruskal's lambda values for null models of network connectivity**. These correlations between the rankings are used to construct dendrograms for each type of null model. Dendrograms illustrate the similarities between centralities (*i.e*., when protein orderings measured with two indices are similar, and their relationships with other centralities do not differ, these two indices are grouped into the same cluster). Click here for file
