# Supplementary material for: Model-based clustering reveals vitamin D dependent multi-centrality hubs in a network of vitamin-related proteins
Source: BMC Syst Biol. 2011 Dec 2;5:195. doi: 10.1186/1752-0509-5-195 (PMC3264545; doi:10.1186/1752-0509-5-195)
Supplement: Additional file 4 — For the 200 vitamin-related proteins we tested whether the number of interactions is determined by the number of publications associated with a given vitamin. We found that the structure of the PPI network is independent from the literature. We also analyzed patterns of vitamin associations in four organisms, observing how human differs from mouse, yeast and E. coli. [file 1752-0509-5-195-S4.PDF]

Additional file 4

Statistical analysis

Vitamins and literature

Proteins associated to vitamin D were strongly represented among network hubs. Here we evaluated whether network structure is biased by the number of manuscripts on different vitamin groups. First, we searched for the number of documents about vitamins from different literature databases: Google Scholar (`http://scholar.google.com/`), PubMed (`www.ncbi.nlm.nih.gov/pubmed/`), Science Direct (`www.sciencedirect.com`) and ISI Web of Knowledge (`www.isiwebofknowledge.com`; see Table S4.1). In the literature, a highly skewed distribution characterizes the number of publications corresponding to the 13 vitamins (null hypothesis = uniform distribution; χ^2, $p \ll 0.001$).

Table S4.1: **Publications on vitamins**

Vitamin	Google	PubMed	ScienceDirect	ISI
A	19,100	46,837	6,777	57,361
B1	11,700	13,946	2,216	19,564
B2	9,250	13,742	2,214	15,745
B3	3,700	7,332	1,100	10,826
B5	3,310	3,695	559	5,030
B6	8,710	11,063	1,472	15,996
B7	9,140	24,942	7,016	43,922
B9	20,300	40,715	6,419	58,757
B12	14,700	25,080	1,532	22,673
C	19,900	52,166	10,035	105,617
D	17,100	42,956	9,196	77,403
E	22,500	36,952	10,801	81,503
K	7,380	14,123	2,292	21,429

We accessed these Websites on October 20th, 2011. Searches were performed for all years, adopting specific keywords (*e.g.*, "vitamin A" or retinol) and with different options: ISI - "topic"; Google Scholar - "title"; ScienceDirect - "abstract, title, keywords"; PubMed - "all fields".

We refined our analysis by collecting the number of publications related to the 200 initial proteins used as a reference for constructing the vitamin PPI network (Additional file 1, last column). These data were extracted from UniProt

(`www.uniprot.org`) and refer to manuscripts indexed by PubMed and/or Medline (the choice of UniProt is coherent with the fact that vitamin-proteins were classified with this dataset). We observed an uneven vitamin association (null hypothesis = uniform distribution; two-sided Kolmogorov-Smirnov test, 13 groups; $D = 0.539,\ p = 0.046$) that is proportional to the relative importance of vitamin groups in the literature (null hypothesis = equal distributions; two-sided Kolmogorov-Smirnov test, 13 groups; $D = 0.154,\ p = 0.998$).

Finally, we compared the number of direct interactions involving the vitamin-proteins (*i.e.*, degree) to the number of publications. A significant difference characterizes the comparison between literature records and protein degrees (two-sided Kolmogorov-Smirnov test; $D = 0.505,\ p \ll 0.001$). Thus, the architecture of the PPI network (*i.e.*, the number of interactions) does not reflect the distribution of protein-related manuscripts. Since centrality scores depend on the network structure, we conclude that our results are not affected by the distribution of manuscripts on vitamin-proteins.

Vitamin-proteins in human and other organisms

The PPI network was constructed with human-specific data. We tested whether vitamin associations for proteins vary between species. Apart from data on human (*Homo sapiens* - 200 proteins) we collected vitamin-related proteins for three organisms that are representative of three key model species: *Mus musculus* (Additional file 5 - 130 proteins), *Saccharomyces cerevisiae* (Additional file 6 - 50 proteins) and *Escherichia coli* (Additional file 7 - 598 proteins). We gathered these data from UniProt and statistical differences were measured with one-sided Kolmogorov-Smirnov tests. Patterns observed for human are significantly different from mouse ($D = 1.000,\ p = 0.003$), yeast ($D = 1.000,\ p = 0.003$) and *E. coli* ($D = 0.833,\ p = 0.016$), in case of six classes of vitamins. Still with six vitamin groups, mouse deviates from yeast ($D = 0.833,\ p = 0.016$) but not from *E. coli* ($D = 0.667,\ p = 0.070$); no statistical difference exists between yeast and *E. coli* ($D = 0.500,\ p = 0.221$). These results vanished in case of 13 groups of vitamins ($p \gg 0.05$). In general, human and mouse are enriched with respect to vitamins A, C, D and K, while the majority of yeast and *E. coli* proteins are associated to vitamins of the group B (Figure S4.1). We conclude that the findings of our study are highly specific to human.

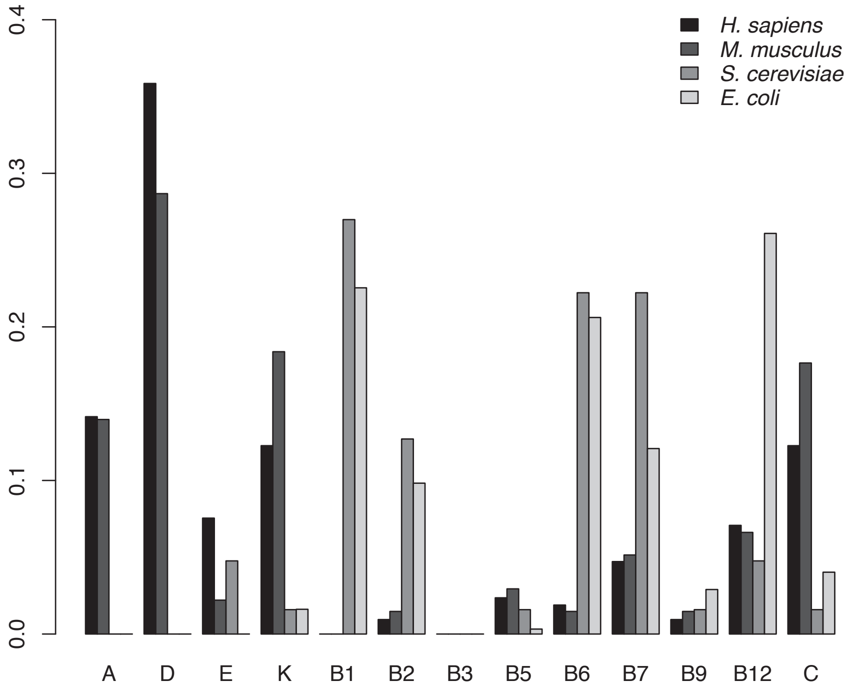

Figure S4.1: **Relative distribution of vitamin-proteins in human, mouse, yeast and *E. coli*.**
