# Supplementary material for: Model-based clustering reveals vitamin D dependent multi-centrality hubs in a network of vitamin-related proteins
Source: BMC Syst Biol. 2011 Dec 2;5:195. doi: 10.1186/1752-0509-5-195 (PMC3264545; doi:10.1186/1752-0509-5-195)
Supplement: Additional file 8 — Correlation matrices describing the Goodman-Kruskal's lambda values for null models of network connectivity. These correlations between the rankings are used to construct dendrograms for each type of null model. Dendrograms illustrate the similarities between centralities (i.e., when protein orderings measured with two indices are similar, and their relationships with other centralities do not differ, these two indices are grouped into the same cluster). [file 1752-0509-5-195-S8.PDF]

Additional file 8

Goodman-Kruskal's lambda

Null models

We generated 4,000 networks evenly distributed between four main types of null models. First, 1,000 networks were obtained by randomly rewiring interactions of the vitamin PPI network, while preserving the degree distribution. Second, we constructed 1,000 random networks according to the Erdős-Rényi model [1]. Random networks had the same number of proteins (1,657) and interactions (2,672) of the vitamin PPI network. Interactions were uniformly and randomly chosen from the set of all possible interactions, with self-loops not allowed. Third, 1,000 small-world networks were created with the Watts-Strogatz model [2]. For obtaining small-world networks we started from a regular lattice of size 668 and with 2,672 interactions. Then we uniformly rewired the interactions with probability $p = 0.05$. Fourth, we generated 1,000 scale-free networks adopting the Barabási-Albert model [3]. This method makes use of a stochastic algorithm, based on a discrete time step mechanism. We preserved the same number of proteins of the vitamin PPI network, while the number of interactions is 1,656. For each simulated network we computed 6 centralities (degree, eigenvector scores, topological importance up to 1 and 4 steps, betweenness and closeness). We normalized the centrality values by setting the maximum to one, and used their scoring for ranking the proteins. Based on these rankings we estimated the Goodman-Kruskal's lambda [4] to extract, for each type of null model, the average correlations between centralities (*i.e.*, we aimed at defining which indices were providing similar protein rankings, to identify potential redundancies). Lambda values for null-models are summarized in Tables S8.1-S8.4; similarities between centralities are showed by the dendrograms of Figure S8.1.

Table S8.1: **Matrix of Goodman-Kruskal's lambda values; rewired networks**

	D	EC	TI^1	TI^4	B	C
D	1.000	-	-	-	-	-
EC	0.515	1.000	-	-	-	-
TI^1	0.715	-0.109	1.000	-	-	-
TI^4	0.960	0.138	0.348	1.000	-	-
B	0.932	0.432	0.670	0.907	1.000	-
C	0.581	0.762	-0.141	0.254	0.543	1.000

Correlation matrix based on Goodman-Kruskal's lambda values. These values were obtained by comparing protein orderings measured for 6 centralities in 1,000 rewired networks. Rewired networks were assembled by preserving size and degree distribution of the vitamin PPI network. Correlations were used for clustering the indices and constructing the dendrogram of Figure S8.1a.

Table S8.2: **Matrix of Goodman-Kruskal's lambda values; random networks**

	D	EC	TI^1	TI^4	B	C
D	1.000	-	-	-	-	-
EC	0.684	1.000	-	-	-	-
TI^1	0.796	0.317	1.000	-	-	-
TI^4	0.924	0.395	0.834	1.000	-	-
B	0.941	0.639	0.563	0.693	1.000	-
C	0.726	0.876	0.332	0.412	0.688	1.000

Based on the protein rankings obtained with the 6 centralities, we estimated the Goodman-Kruskal's lambda values. This correlation matrix refers to 1,000 random networks with the same size of the vitamin PPI network. Lambda values allowed to estimate the dissimilarity structure between indices (see Figure S8.1b).

Table S8.3: **Matrix of Goodman-Kruskal's lambda values; small-world networks**

	D	EC	TI^1	TI^4	B	C
D	1.000	-	-	-	-	-
EC	0.598	1.000	-	-	-	-
TI^1	0.969	0.197	1.000	-	-	-
TI^4	0.990	0.147	0.891	1.000	-	-
B	0.595	0.246	0.340	0.388	1.000	-
C	0.405	0.294	0.193	0.219	0.567	1.000

Goodman-Kruskal's lambda values describing the rank correlation between protein orderings. Orderings were computed using the 6 centralities in 1,000 small-world networks with the same number of interactions of the vitamin PPI network, but smaller size (668 proteins). Outcomes were used to construct a dendrogram which identifies redundant information portrayed by certain centrality indices (see Figure S8.1c).

Table S8.4: **Matrix of Goodman-Kruskal's lambda values; scale-free networks**

	D	EC	TI^1	TI^4	B	C
D	1.000	-	-	-	-	-
EC	0.221	1.000	-	-	-	-
TI^1	0.978	-0.078	1.000	-	-	-
TI^4	1.000	-0.100	0.848	1.000	-	-
B	0.985	0.220	0.915	0.940	1.000	-
C	0.231	0.880	-0.078	-0.108	0.231	1.000

Goodman-Kruskal's lambda values for correlations between protein rankings. Correlations refer to 6 centralities measured in 1,000 scale-free networks with the same size of the vitamin PPI network, but with 1,656 interactions. Data show which indices were providing similar protein rankings and, based on these outcomes, centralities were clustered. The dissimilarity structure between centralities is illustrated in the dendrogram of Figure S8.1d.

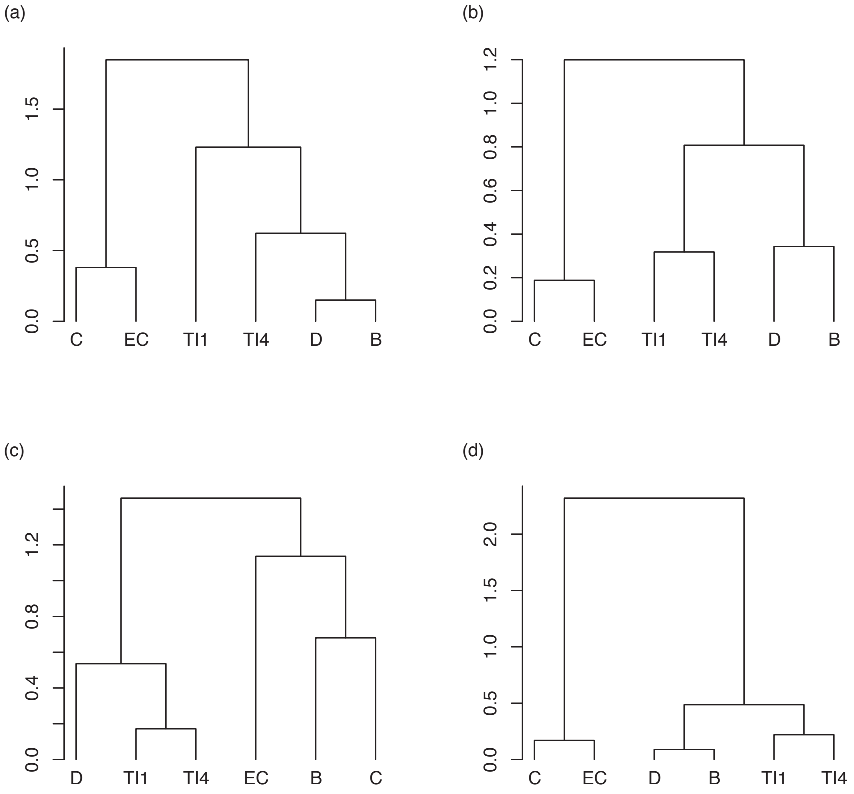

Figure S8.1: **Dendrograms based on centrality scores in null models.** Using the 6 centralities that were measured in null models we estimated Goodman-Kruskal's lambda values. They quantify the correlations between each pair of centrality. Cluster composition depends on the correlations listed in Tables S8.1-S8.4. Centralities that are grouped into the same cluster are redundant. Ranking the proteins with centralities that are included into the same cluster does not provide different orderings. For each null model, we identified the main clusters and their composition changes according to model types: (a) rewired networks, (b) random networks, (c) small-world networks, (d) scale-free networks. Closeness and eigenvector scores tended to be grouped together. Degree and betweenness provided similar protein rankings, except for the case of small-world networks. Topological importance indices were always clustered together, although topological importance up to 1 step clearly deviates from this pattern in rewired networks.

References

[1] Erdős P, Rényi A: **On random graphs**. *Publicationes Mathematicae* 1959, **6**:290–297.

[2] Watts DJ, Strogatz SH: **Collective dynamics of 'small-world' networks**. *Nature* 1998, **393**:440–442.

[3] Barabási AL, Albert R: **Emergence of scaling in random networks**. *Science* 1999, **286**:509–512.

[4] Goodman LA, Kruskal WH: **Measures of association for cross classifications**. *Journal of the American Statistical Association* 1954, **49**:732–764.
